# The Centre for Data and Knowledge Integration for Health (CIDACS): Linking Health and Social Data in Brazil

**DOI:** 10.23889/ijpds.v4i2.1140

**Published:** 2019-11-20

**Authors:** ML Barreto, MY Ichihara, BA Almeida, ME Barreto, L Cabral, RL Fiaccone, RP Carreiro, CAS Teles, R Pitta, GO Penna, M Barral-Netto, MS Ali, G Barbosa, S Denaxas, LC Rodrigues, L Smeeth

**Affiliations:** 1 Centre for Data and Knowledge Integration for Health (CIDACS), Gonçalo Moniz Institute, Oswaldo Cruz Foundation (FIOCRUZ), Salvador, Brazil.; 2 Institute of Collective Health, Federal University of Bahia (UFBA), Salvador, Brazil.; 3 Computer Science Department, Federal University of Bahia (UFBA), Salvador, Brazil.; 4 Statistics Department, Federal University of Bahia (UFBA), Brazil.; 5 Tropical Medicine Centre, University of Brasília (UnB), Brazil.; 6 Escola Fiocruz de Governo, FIOCRUZ Brasília, Brazil.; 7 Center for Statistics in Medicine, Nuffield Department of Orthopaedics, Rheumatology and Musculoskeletal Sciences, University of Oxford, Oxford, UK.; 8 Faculty of Epidemiology and Population Health, London School of Hygiene and Tropical Medicine, United Kingdom.; 9 Institute of Health Informatics, University College London, United Kingdom.

## Abstract

The Centre for Data and Knowledge Integration for Health (CIDACS) was created in 2016 in Salvador, Bahia-Brazil with the objective of integrating data and knowledge aiming to answer scientific questions related to the health of the Brazilian population. This article details our experiences in the establishment and operations of CIDACS, as well as efforts made to obtain high-quality linked data while adhering to security, ethical use and privacy issues. Every effort has been made to conduct operations while implementing appropriate structures, procedures, processes and controls over the original and integrated databases in order to provide adequate datasets to answer relevant research questions. Looking forward, CIDACS is expected to be an important resource for researchers and policymakers interested in enhancing the evidence base pertaining to different aspects of health, in particular when investigating, from a nation-wide perspective, the role of social determinants of health and the effects of social and environmental policies on different health outcomes.

## Introduction

The Centre for Data and Knowledge Integration for Health (CIDACS)1https://cidacs.bahia.fiocruz.br/ was created in Salvador, Bahia-Brazil (December 2016), as part of the Oswaldo Cruz Foundation (FIOCRUZ)2https://www.bahia.fiocruz.br/cidacs/, an institution affiliated with the Brazilian Ministry of Health. CIDACS aims to conduct interdisciplinary research, develop new scientific methodologies and promote professional training using linked large-scale databases and high-performance computational resources in a secure environment.

The use of administrative data for research is at the core of CIDACS’ activities. Administrative data comprises information on individuals that was not primarily collected for research purposes but can be prepared and integrated for use in research projects. This data originates from a variety of national government departments and agencies in the process of delivering governmental services, and includes data generated through the administration of government programs (e.g. mortality system data, information on users of social services).

The many advantages of administrative data include large sample sizes, longitudinal structure, high population coverage and high data quality [1]. The use of large-scale databases and data linkage for population health research has been developed in high-income countries, in particular the United Kingdom, Australia and Canada [2]. Some other countries have also developed initiatives to integrate most of their population data, e.g. Denmark [3,4], Switzerland [5] and New Zealand [6]. In Brazil, recent improvements in the coverage and quality of health information systems, extending to the databases thusly generated, have enabled the use of routinely collected data for research.

The creation of a data centre (DATASUS)3http://www2.datasus.gov.br/DATASUS/index.php in 1991 at the Brazilian Ministry of Health to process, store and manage national databases (e.g. deaths, births, hospitalisations, infectious disease notifications, etc.) and to stimulate data access for policymakers and researchers, has made it possible to conduct intensive research. The collected data can be i) grouped on a municipal level (Brazil has 5,570 municipalities), ii) individually de-identified/anonymized or iii) individually identified. Grouped data have been used in a large number of studies, including relevant evaluative studies investigating the impact of the Brazilian cash transfer programme (Bolsa Família) on health outcomes [7-9].

The use of individually de-identified or identified data has presented an opportunity for Brazilian researchers to investigate in great detail the strengths and limitations of a variety of administrative databases.

Several experiences of data linkage in Brazil have been reported focusing on specific issues and the development of record linkage tools, notably Reclink4http://reclink.sourceforge.net/ [10], which was widely used in a series of different linkage-based studies. Pioneering experiences at the Federal Universities of Rio de Janeiro and Minas Gerais have also produced important scientific contributions [11,12]. It is worth noting that before the promulgation of the Information Access Law5http://www.planalto.gov.br/ccivil_03/_ato2011-2014/2011/lei/l12527.htm in 2011, it was relatively easy to obtain access to identified data; however, while it is still possible for researchers to obtain such data nowadays, this process has become much more restrictive and with improved data privacy protection rules.

In Brazil, observational approaches (e.g., case-control and cohort studies) applied to large administrative linked databases have been mostly used to investigate associations between exposures and health outcomes [13,14]. However, the linkage of large administrative databases has rarely been used to evaluate how governmental policies targeting the social determinants of health (e.g., social protection policies) can affect health outcomes.

Accumulated evidence indicates that social, economic and environmental components (e.g., poverty and inequalities) can influence many different aspects of illness and health [15]. While there is substantial evidence on the effects exerted by different social and economic factors on health, little is known on the health impacts of policies designed to affect socioeconomic factors [16,17]. The development of social protection policies, of which cash transfer programmes are an example, has demonstrated not only a capacity to reduce levels of poverty and inequality, but also a strong potential to positively affect health [18]. In Brazil, studies focused on the impact of social protection policies on health outcomes have been designed using ecological (aggregate) approaches [7,19]; however, few studies to date have employed linked individual data [20-22].

The establishment of a data center, as a resource primarily focused on investigating the effects of social protection programmes on health outcomes using databases and proper methodological approaches (e.g. quasi-experiments [16] or natural experiments [23]) was the primary motivation behind the creation of the 100 Million Brazilians Cohort and the construction of CIDACS, where the cohort and its related data are stored, processed and accessed.

## Approach

### Population setting

CIDACS was founded with the aim of establishing the 100 Million Brazilians Cohort, an effort focused on enhancing the understanding of the impact of social protection policies (e.g., cash transfer, housing and cisternsupply programmes) on health outcomes in low-income populations throughout Brazil. The centre’s primary data source is the Unified Registry for Social Programmes (CadUnico) database, which includes individuals eligible to receive benefits from over 20 governmental social protection programmes.

The CadUnico database contains identifiable records with socioeconomic and demographic data, as well as household characteristics pertinent to every person who has applied for any governmental social benefit since 2003. By 2015, more than 114 million people were registered in CadUnico, representing 57% of the entire Brazilian population.

The extensive representation of the CadUnico population base makes it suitable for linkage with other administrative databases, such as those generated by health and education services, conditional cash transfer and housing programmes, in addition to other research databases from a variety of sources. It is worth noting that some of the other databases currently stored at CIDACS, such as those detailing information on deaths, births, notification of infectious diseases and other acute events, cover the entire Brazilian population.

### Operating model

DACS obtains identified data upon request and approval from different government agencies. Obtained data are linked and, upon request,deidentified/anonymized datasetscan can be accessed by researchers working on specific research questions. CIDACS develops and executes data preparation and linkage procedures, as well as implementing quality and consistency measures to assess link age accuracy in aneffort to provide datasets from well-characterized and representative population datasets.

CIDACS possesses two separate environments for data handling. The *Data Production Centre* is a secure room housing the computational infrastructure for ingesting, storing, cleaning, processing and linking original identified databases, and for extracting research-ready de-identified/anonymized datasets. The *Data Analysis Environment* (DAE) is a computational infrastructure that gives researchers access to requested de-identified/anonymized datasets under the terms and conditions established by the centre.

### Architecture and information technology

**Figure 1: The layered structure of the CIDACS data platform.**
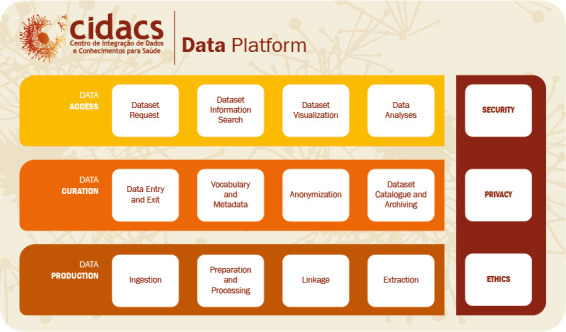


CIDACS has developed a data platform capable of providing data management functions necessary for research projects. The data platform enables the collection, storage, processing and linkage of large amounts of data, as well as data cataloging and the provision of access to large datasets for analysis [24,25]. Conceptually, the data platform (represented in Figure 1) models components involving people, processes and computational resources related to data lifecycles.

Regarding the *data production layer*, original data sources are ingested and processed through a linkage pipeline consisting of preprocessing, the application of linkage algorithms and accuracy evaluation (post-processing). Source data ingestion can also include accumulated database backups made over several years (as in the case of CadUnico).

Data extraction are performed on demand. Upon the receipt of a data plan from a researcher, a dataset combining data from several databases with linked records is built and/or extracted. Anonymisation procedures are applied, including de-identification and the aggregation of sensitive information. CIDACS employs encrypted keys as record identifiers.

The computational infrastructure in this layer consists of a cluster of machines, managed using *Hadoop*6http://hadoop.apache.org/ technology. Data storage is implemented as a data lake, allowing data in different formats to be first stored and structured later. Linkage algorithms are executed using *Spark*7https://spark.apache.org/ in a distributed fashion over cluster nodes. When CIDACS data engineers use this infrastructure, no internet, mobile phone or any other type of external online access is permitted.

In the *data curation* layer, curators verify original databases, approve data plans, catalogue dataset metadata and employ anonymisation procedures. An interface between data production and data access is also provided. We implement a *dataverse*8https://dataverse.org/ repository, providing metadata and *handle*9http://www.handle.net/ identifiers for datasets. The generated metadata provides researchers with dataset documentation, including a data dictionary, descriptive statistics, data preparation procedures and quality measures.

In the *data access* layer, the user completes several forms and access agreements, including a data plan, which is required to request a dataset. Data can be accessed and analyzed in the Data Access Environment (DAE), where custom-built de-identified datasets are made available to researchers. Data can also be further explored using visualization tools.

The DAE environment runs on Blade server infrastructure and is managed as a pool of virtual machines configured for each project. DAE follows the *Data Safe Haven* principles by providing controlled access permissions, controlled data and software entry and exit, and the monitoring of activities (i.e. logs). Data exit from DAE are permitted only in the form of aggregated data suitable for publication.

CIDACS adheres to the FAIR (Findable, Accessible, Interoperable and Reuseable) principles of data management and stewardship [26]. The FAIR principles enable distinctions between data and metadata to support a wide range of special circumstances for data management. CIDACS implements the *findable* principle through its catalogue (dataverse) and the *accessible* principle through its DAE. Security, privacy and ethics are prioritized across all layers via a set of policies and agreements.

### Governance, legislation and management

Initially, the organization of CIDACS was designed to achieve the objective of creating the 100 Million Brazilians Cohort. Due to the expansion of the project’s initial scope, as well as the incorporation of new studies and additional partnerships, CIDACS has reconfigured its organizational arrangements accordingly. Consequently, the centre’s governance structure has adopted a flexible and dynamic management model that is both cross-sectoral and systemic. Based on individual and collective interdisciplinary competences, its system of governance deals with scientific and technical-operational issues to generate strategic and social value.

In addition to the institutional resources allocated to CIDACS by Fiocruz to cover basic administrative maintenance, funding has also been assured through resources from national and international agencies interested in supporting investigations into social determinants of health, evaluating the impact of social policies on the overall health of the Brazilian population and other population health-related issues.

The CIDACS team is comprised of a small group of Fiocruz employees who possess research experience in diverse areas of public health, research collaborators from different national and international institutions, in addition to hired professionals with expertise in operational areas (data production, project management, data curation) and postdoctoral researchers.

**Figure 2: CIDACS’ Governance Structure:**
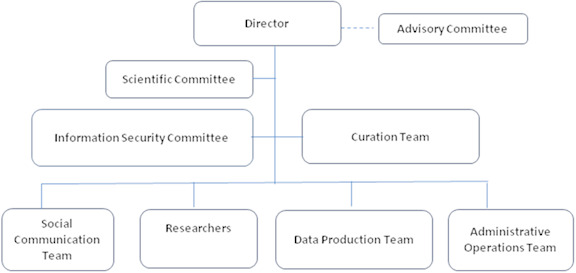
- Director: supported by an Advisory Committee: responsible for carrying out the institution’s strategic plan, including follow-up and recommendations;
- Scientific Committee: evaluates compliance with the requirements for conducting research in line with CIDACS’ mission and in accordance with ethics regulations;
- Information Security Committee: provides advice on policies and the implementation of data protection strategies;
- Data Curation Team: responsible for managing the lifecycle of all databases (administrative, scientific and others) obtained by or produced within CIDACS in order to maintain, preserve and add value;
- Social Communication Team: uses strategies to divulge the knowledge produced by CIDACS and other related topics to researchers, managers, policymakers and society in general, in addition to promoting internal communication;
- Researchers: responsible for carrying out individual research projects or collaborating on larger projects. This includes CIDACS researchers and external researchers across Brazil and internationally;
- Data Production Team: responsible for performing data processing procedures (preprocessing, linkage, accuracy verification and extraction);
- Administrative Operations Team: performs all actions related to infrastructure maintenance, logistics, human resources and project management.

CIDACS’ governance framework handles scientific and technical-operational issues by creating environments that enable the production of multidisciplinary knowledge. The centre also actively disseminates the knowledge generated to other organizations. Its governance structure consists of different and complementary areas, as illustrated in Figure 2.

CIDACS’ governance principles seek to prioritize the privacy of individuals, as well as collective rights and interests, in addition to the quality of linked datasets. Our centre strives to ensure that management decisions are made in a transparent, safe, responsible and inclusive manner.

### Consent model

In Brazil, until the passage of Federal Law 12.527/2011 (the Information Access Law), no national laws regulated the access to information collected by government institutions. Under this law, the access to administrative data containing personal information for research purposes first requires ethical approval by institutional review boards, followed by formal authorization from government data system administrators. All research involving personal data requires the explicit approval of institutional review boards (CEPs), which together with the National Research Ethics Committee (CONEP) form the CEP/CONEP system. Whenever individual consent is not feasible, ethical assessments are focused on risk-benefit analysis, participants’ rights, measures to prevent harm to and discrimination of individuals or groups, researcher responsibilities and the monitoring of approved research projects10http://conselho.saude.gov.br/resolucoes/2012/Reso466.pdf. Accessed May, 2019..

The Brazilian General Data Protection Law was passed in 2018 (Law 13.709/2018), and will take effect in August 2020. This legislation, which was largely inspired by the European Union General Data Protection Regulation11https://eugdpr.org/, creates positive conditions for the use of personal data for research purposes by establishing a series of principles and privacy protection measures. It also contemplates the use of such data for public health research (see 13th article). This law establishes that any individual whose data is stored in a database can request, from the data guardian, at any time, information regarding the sharing of his/her personal data, the criteria and procedures used in the treatment of personal data and request the removal (opt-out) of his/her data from the database in question.

### Privacy by design

CIDACS’s data management structure has been designed to preserve confidentiality through a combination of physical and virtual settings to guarantee the privacy of identifiable data and also to restrict the possibility of re-identification of de-identified data. This policy is in accordance with guidelines for safe data linkage, including the separation of linkage and analysis processes [27]. All data procedures include physical and virtual security measures, which are capable of preventing unauthorised access to the identified databases, as well as linked de-identified/anonymized datasets. All individuals involved in handling or analyzing data are also made aware that legal action will be taken if data are used improperly or without the appropriate care necessary to protect privacy.

### Databases

To date, CIDACS hosts several databases from government administrative systems, which are summarized in Table 1. The size indicates the number of records after preprocessing.

**Table 1: Administrative databases hosted at CIDACS.** 

Databases	Description

Unified Registry (Cadastro Único – CadUnico) (2001 - 2018)	114,008,317 records between 2001-2015. National Register for social benefits. Data from 2016 to 2018 are currently being processed. National coverage. Database includes data on families and individuals applying the several social protection programmes. Individuals may be eligible for different social benefits. Data includes demographics, family composition, income, work, education, expenses, housing type, water supply, sanitation, electricity, garbage collection, among others. Data are updated whenever essential information changes, e.g. household composition, school and income, or every two years to update beneficiary records.

Cash Transfer Programme (Bolsa Família – PBF) (2004 -2015)	27,376,582 families. National coverage. Database includes data on families and individuals receiving benefits from the Brazilian conditional cash transfer programme. Datapoints include start and end date of benefit endowments, total value received per family, participant age, number of months of received payments.

Housing Programme (Minha Casa Minha Vida) (2009-2017)	1,509,749 records. National coverage. Database includes families and individuals receiving benefits from the Housing Programme for poor families. Housing units are offered based on income. Data include benefits received and family composition of beneficiaries.

Cistern Supply Programme (2011-2016)	1,002,375 records. Rainwater collection cistern distribution programme. Semi-arid zone coverage, including information on municipality of residence, state, sex, schooling, income, number of persons in family, sanitation, water consumption and treatment, housing type.

Mortality Information System (SIM) (2000 -2017)	20,510,101 records. National coverage. Database includes information on individuals at time of death, including maternal and child information. Data are recorded on type of death, date of death, date of birth, gender, race, education, duration of pregnancy, single or multiple pregnancies, type of delivery, age of mother, gestational age, birth weight and cause of death.

Live Births Information System (SINASC) (2001-2017)	50,265,232 records. National coverage. Database includes information on individuals at time of birth, including maternal information. Data include mother’s date of birth, education, marital status, number of children and abortions, number of prenatal consultations, date of last menstruation, gestational age, type of pregnancy, type of delivery, child’s date of birth, gender, race, birth weight, presence of anomaly, who attended the childbirth.

Disease Notification Information System (SINAN) (2001-2016)	The original source contains notifications on 52 different diseases, comprised of several separate databases for each disease. National coverage. Currently, CIDACS hosts the following databases: SINAN Tuberculosis (2001-2013): 1,114,740 records SINAN Hansen (2007-2016): 771,039 records SINAN Zika (2013-2016): 309,796 records SINAN Dengue (2007-2016): 13,222,763 records SINAN-Chikungunya (2012-2016): 303,523 records

Register of Infectious Events in Public Health (RESP- microcephaly)	16,242 records. National coverage. Database contains data on microcephaly notification, including microcephaly cases, mother’s clinical and epidemiological data, ethnicity, skin color, location of residence, child’s sex, birth date, birth weight, length and information regarding delivery, pregnancy events.

Hospitalization Information System (SIH) (2000-2017)	The original source contains hospitalization information on individuals admitted to public or private hospitals whose hospital stays were covered by SUS. State-level coverage. CIDACS is in the process of obtaining all national data. Currently, the following databases have been preprocessed: SIH Bahia State (2000-2017): 9,758,611 records SIH MG State (2002-2017): 8,438,288 records

Food and Nutrition Surveillance System (SISVAN) (2008-2017)	The original source contains nutritional information on individuals. National coverage. Database includes data on anthropocentric indices, food consumption, age and municipality of residence. Currently, the following databases have been processed: SISVAN- Anthropometric data (2008-2017): 97,156,600 records SISVAN- Food consumption data (2015-2017):2,055,674 records.

### Data linkage

**Figure 3: Standard data linkage flow at CIDACS:**
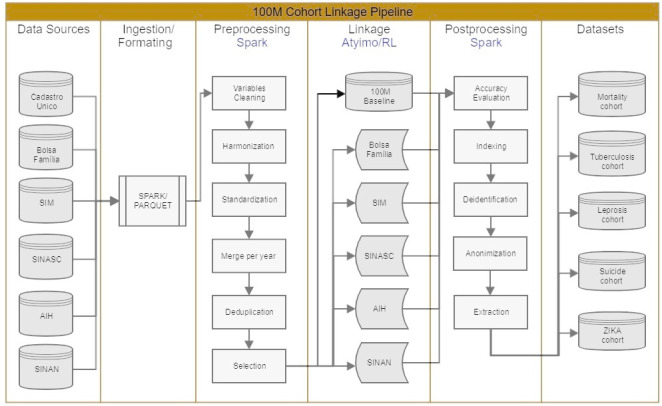


CIDACS has two distinct linkage scenarios: *deterministic*, involving social programme databases, and *probabilistic*, involving health databases. The 100 million Brazilians cohort is the result of several linkages between social and health databases (Figure 3). Following the ingestion and formatting of the original databases, the pipeline involves three main steps: preprocessing, linkage (matching algorithms) and post-processing.

Preprocessing is an intensive procedure conducted in each original database. It consists not only of preparing linkage variables, but it also includes the input of epidemiologists and statisticians to ensure a useful data format for research purposes. When necessary, consultations with source providers are performed to clarify variables or questions about the format used. Operationally, preprocessing involves the cleaning, standardization, harmonization and merging of data, as well as deduplication, selection or derivation of variables deemed useful for research.

The application of a linkage algorithm generates a dataset containing the identification and matching scores between the individuals from the two databases involved. The accuracy assessment procedure provides a cut-off point above which these individuals are considered identical (linked) and below which they are not identical (not linked). Resulting linked datasets are stored and used to build research cohorts.

Accuracy assessment consists of acquiring samples (n=2000) from each linked dataset for manual evaluation and measuring linkage quality based on metrics, such as specificity and sensitivity and the area under the ROC curve at selected points (candidate cut-off points). [28] The final cut-off point is considered that which maximizes the measures above.

To support data linkage, CIDACS has developed two different tools adapted to the type and size of the databases to be linked. These tools differ mainly in the blocking step used to select records for matching. Atylmo [29] was designed for deterministic and probabilistic linkage. Blocking is performed through a set of predicates that select records into blocks. Bloom filters [30,31], which transform bigrams from the linkage key attributes into a binary vector, are used for similarity calculation (matching). Atylmo has proven to be quite effective, providing 93% to 97% of accuracy (true positive rate) depending on the databases being linked [32]. CIDACS-RL, another linkage tool designed over Apache Lucene12https://lucene.apache.org/, uses a novel approach based on an indexing search and sorting algorithm to perform information retrieval. TF-IDF (term frequency – inverted document frequency) [33] is used as a blocking procedure to reduce the number of comparisons during linkage. Queries can be of three types (exact, semi-exact or fuzzy), allowing for different similarities (scores) when performing deterministic linkage using numerical and categorical attributes. Sorting is then performed to retrieve the most similar records following a determined cut-off value. CIDACS-RL was internally tested over large databases, with a very high resulting accuracy (96% of true-positive pairs).

Deterministic linkage with CadUnico occurs for databases related to social programmes through a common unique key: Social Identification Number (NIS). In order to receive benefits from any Brazilian governmental social programme, an individual must first register with CadUnico and receive a NIS. Therefore, this attribute can be used to link demographic and socioeconomic data recorded in CadUnico with data from the specific social programme from which an individual has received benefits (e.g., Cash Transfer or Housing programs).

Probabilistic linkage with CadUnico is applied to health databases when there are no common unique keys. In such cases, a set of attributes commonly present in all health databases as well as in CadUnico is used: name, gender, mother’s name, date of birth and municipality code.

For the 100 Million Brazilians Cohort13https://cidacs.bahia.fiocruz.br/en/platform/cohort-of-100-million-brazilians/ several linkage strategies were needed to bring the maximum number of individuals into the cohort. For example, the linkage of living births (SINASC database) with CadUnico involved two stages: in the first stage, mothers were probabilistically linked to individuals in CadUnico, whereas, in the second stage, children were deterministically linked based on their mother’s information, since newborn children might not have received names at the time of registration.

A recent effort has been to georeference or geocode this data. Some data have already been transferred with this information (CadUnico), while geocoding is under development for other data (SIM and SINAN).

### Data access

Currently, only researchers and authorized personnel from government agencies and national and international research institutions that collaborate with CIDACS can access linked and de-identified/anonymized data. These individuals and organizations must be committed to advancing scientific knowledge or generating evidence for the formulation of public policies. Researchers are permitted to exclusively access de-identified/anonymized data specifically relevant to their proposed study objectives.

Persons who wish to receive authorisation must:

- Be affiliated to the institution or be identified as collaborators;
- Present a detailed research project together with ethical approval by an appropriate Brazilian institutional review board;
- Provide a clear data plan restricted to the objectives of the proposed study, and a summary of the analysis plan intended to guide the linkage and/or extraction of a relevant set of records and variables;
- Sign terms of responsibility regarding the access and use of data;
- Perform the analysis of datasets provided using the CIDACS data analysis environment, a safe and secure infrastructure that provides remote access to de-identified/anonymized datasets and analysis tools.

### Noteworthy outputs

At the time of writing, approximately 30 studies are being conducted using linked data sources, including nation-wide studies on the social determinants and the impact of conditional cash transfer and housing programs for low-income populations, on different health outcomes, such as tuberculosis, leprosy, cardiovascular diseases, child and maternal health, suicides and homicides. The first two manuscripts reporting results from the cohort analysis were recently published, while others are under review or preparation. The first reports on the social determinants of leprosy [34], while the second [35] investigates leprosy treatment default.

With the support of the Ministry of Health, the research project entitled *Long-term Zika virus and microcephaly surveillance in the Brazilian Universal Health System (SUS)* aims to follow the long-term effects of Zika infection in Brazilian children over an extended period. This will entail the integration of data from the national birth registry (SINASC) linked to health, social and educational information for long-term monitoring. This should enable a better understanding of morbidity and mortality related to Zika virus infection and its consequences, and is designed to enhance scientific knowledge surrounding this disease and support the adoption of public health measures.

The centre is participating in the first call of the *Grand Challenges Explorations - Brazil: Data Science Approaches to Improve Maternal and Child Health in Brazil* 14This call was conceived by the Bill & Melinda Gates Foundation, the Brazilian Ministry of Health and the Brazilian Research Council. Call available at: https://gcgh.grandchallenges.org/challenge/grand-challenges-explorations-brazil-data-science-approaches-improve-maternal-and-child. The role of CIDACS is to provide de-identified/anonymized datasets derived from the 100 million Brazilian cohort, as well as the necessary analysis infrastructure to selected applicants. This pilot experience is expanding the number of authorized users who are granted access to CIDACS de-identified/anonymized datasets, as well as allowing CIDACS to mature its procedures pertaining to data production, access and protection for external users.

## Discussion and conclusions

Over the years, a large community of public health researchers in Brazil have used national health and social databases for research purposes. The increasing use of linked data represents an advance that allows investigators to address questions that would be otherwise infeasible through the use of disparate databases [10,12,22,36].

CIDACS is a result of the efforts of researchers, policymakers and public health officials who aim to use and improve the quality of Brazilian health databases. The centre strives to be a resource for researchers and policymakers interested in improving the quality of evidence on different aspects of health, in particular the social determinants of health and the effects of social and environmental policies on health. Additionally, it has already demonstrated its potential to contribute to other health challenges, such as the Zika study referred to above, as well as evaluating the effectiveness and side effects of health technologies used in the Brazilian Unified Health System [37].

The fact that CIDACS houses very large datasets linked in the form of cohorts presents considerable advantages. First, the linkage of health and social data originating from various government sectors adds enormous value to already-existing health data, allowing for the study of the determinants of both drivers of health and the consequences of ill health. Second, it allows for the study of the poorest populations in Brazil, especially from 2011 onwards, after which registration of extremely poor families in the CadUnico system was intensified, with coverage expanded on a national level. Third, the longitudinal structure of the data used is methodologically sound, enabling the addition of new exposures or outcomes, the study of outcomes at different times of exposure, including over the long-term, and the evaluation of various social protection policies on health outcomes. Fourth, large sample sizes enable the analysis of small groups and rare events in ways not possible in projects dependent on the collection of new primary data [14,38]. Fifth, linkage is conducted with powerful and accurate software developed in-house (AtyImo and CIDACS-RL). Sixth, the datasets generated by CIDACS hold the potential for use in other areas. For instance, it is possible to add other variables (e.g. death) to surveys or traditional cohorts, as well as to examine outcomes directly collected by surveys (e.g. nutritional status) in evaluative studies on the impact of social protection policies.

As the existing databases and cohort are of unprecedented size, efforts have been made to set up the necessary infrastructure and develop associated tools for an increasing number of researchers working in a wide range of health-related areas to utilize these data sources.

It is worth noting that CIDACS members are also participating in discussions at public meetings, as well as talking with different stakeholders regarding the privacy and ethical aspects associated with the use of large administrative databases for research and policy evaluation.

Several issues continue to challenge CIDACS, including keeping linked datasets up-to-date, performing data curation in a timely manner, and maintaining and expanding computational resources to optimize data production and analysis. It is also necessary to provide training for researchers on the manipulation and analysis of large datasets, a task not feasible using traditional methods, tools or computational environments. We also recognize the need to inform data users regarding the alignment of data requests to the specific research questions due to privacy concerns.

Based on our practical experiences to date, CIDACS is currently revising its institutional policies related to data governance as well as other aspects related to data access, use and reuse, with the objective of being fully compliant with the Brazilian General Data Protection Law, which will take effect in August 2020, in addition to any other related new procedures as required by the National Research Ethics Committee.
